# Supplementary material for: Dynamic and thermodynamic influences on precipitation in Northeast Mexico on orbital to millennial timescales
Source: Nat Commun. 2023 Apr 20;14:2279. doi: 10.1038/s41467-023-37700-9 (PMC10119167; doi:10.1038/s41467-023-37700-9)
Supplement: Supplementary file 1 — Supplementary Information [file 41467_2023_37700_MOESM1_ESM.pdf]

Supplementary Information for:

Dynamic and thermodynamic influences on precipitation in NE Mexico on orbital to millennial timescales

Kevin T. Wright[1*], Kathleen R. Johnson[1*], Gabriela Serrato Marks[2], David McGee[2], Tripti Bhattacharya[3], Gregory R. Goldsmith[4], Clay R. Tabor[5], Jean-Louis Lacaille-Muzquiz[6], Gianna Lum[1], Laura Beramendi-Orosco[7]

*Corresponding authors: ktwright@uci.edu (K.T.W.); kathleen.johnson@uci.edu (K.R.J.)

This .docx file includes:

The attached supplementary information .docx file includes:

Supplementary Notes 1-11
Fig. S1. Modern precipitation patterns of NE Mexico.
Fig. S2. Isotopic composition of precipitation.
Fig. S3. Cave temperature, relative humidity from 2017-2020.
Fig. S4. Proxy system modelling of speleothem $\delta^{18}O$.
Fig. S5. CB2 $\delta^{18}O$ and $\delta^{13}C$ with age uncertainties.
Fig. S6. CB2 $\delta^{18}O$ compared to various seasons of insolation.
Fig. S7. CB2 $\delta^{18}O$ compared to regional SSTs.
Fig. S8. iCESM1 precipitation and rainfall data compared to observations.
Fig. S9. Moisture budget analysis of precipitation change.
Fig. S10. Summer and winter low level winds during the Last Glacial Maximum, Mid-Holocene and Pre-Industrial Period.
Fig. S11. Summer and winter precipitation during the Last Glacial Maximum, Mid-Holocene and Pre-Industrial Period.
Fig. S12. Precipitation and soil-moisture comparison between the Pre-Industrial to Last Glacial Maximum.
Fig. S13. Radiocarbon bomb peak in a modern speleothem.
Fig. S14. CB2 age model with various initial $^{230}Th/^{232}Th$ values and uncertainties.
Fig. S15. Comparison of raw CB2 $\delta^{18}O$ with mean-seawater subtracted CB2 $\delta^{18}O$.
Table S1. Uranium-thorium data for Stalagmite CB2.
Table S2. Radiocarbon data for a modern Cueva Bonita speleothem sample.
Table S3. Matching various initial Th to the bomb peak in a modern cave sample.
Table S4: Precipitation from Alta Cima, Mexico and cave water $\delta^{18}O$ and δD data.

Supplementary Note 1

Stalagmite CB2 was collected on a slope approximately 30 meters from the cave entrance on a second level, approximately 15 meters below the first level. Stalagmite CB2 was inactive when collected, but drip water was collected from around the sample in spring 2016 and 2017. CB2 is a 78 cm long stalagmite composed of translucent grey and beige calcite. No acicular patterns were detected by hand lens, and XRD analysis conducted at ~ 15cm intervals were confirmed samples were all calcite. Based off the appearance of the sample, we suggest the entire stalagmite is calcite. CB2 exhibits a predominantly aggregational stratigraphic stacking pattern as well as a globular morphostratigraphic units throughout, suggesting relatively consistent drip rates and calcite saturation (Martín-Chivelet et al., 2017).

Supplementary Note 2

Cueva Bonita (23°N, 99°W; 1071 m above sea level) is located in the highlands of the Sierra Madre Oriental in the northeast Mexican state of Tamaulipas (Fig. S1). Regional geology is dominated by thick (>2000 m) Lower Cretaceous limestone (Gary and Sharp, 2006), uplifted by normal and thrust faults (Ford, 2000). The cave has ~20-25 m of overburden, a single relatively narrow entrance, and a shallow soil profile with 30 cm of dark brown clay loam soil. The vegetation above the cave is dominated by C_3 plants, including the *Podocarpus reichei*, *Liquidambar styraciflua*, *Quercus sartorii*, *Quercus germana*, *Clethra pringlei*, *Magnolia tamaulipana*, *Acer skutchii, Cercis canadensis*, and diverse epiphytic orchid species. The cave resides within the northernmost tropical cloud forest, with an annual mean precipitation of 1800 mm/yr (Grams & Faaborg, 1997) and annual mean exterior temperature of 23.3°C (Fig. S1).

Supplementary Note 3

An ongoing cave monitoring campaign has been conducted to better understand how the external climate is recorded in speleothem calcite. Relative humidity and temperature fluctuations have been monitored using an Onset HOBO Relative Humidity-Temperature logger, collecting data every 2 hours. Additionally, daily CO_2 measurements were made using a handheld Vaisala GM-70 CO_2 monitor attached to a 12 Volt lantern battery, which typically lasted 1-3 months. Over a 27-month period, relative humidity remained constant at 100% (Fig. S3). The temperature varied only slightly from the mean of 17.5°C with a subtle drop (< 0.5°C) during boreal winter (Fig. S3). These stable conditions promote calcite deposition in near isotopic equilibrium conditions and calcite $\delta^{18}O$ is therefore reflective of the isotopic composition of precipitation. Monitoring revealed a seasonal cycle in cave pCO_2, ranging from atmospheric concentrations (~400 ppm) during winter, upwards to 1251 ppm during the warmer summer months (Fig. S3). This change in pCO_2 suggests there may be a seasonal cycle in speleothem $\delta^{13}C$ as has been shown in other studies on the effects of changing cave ventilation on speleothem chemistry (Frisia et al., 2011). This ventilation related fractionation is not likely to significantly impact speleothem $\delta^{13}C$ variations on interannual timescales or longer.

Supplementary Note 4

During periods of reduced local water balance (i.e. reduced water in the critical zone, P-ET), Prior Calcite Precipitation is enhanced due to an increase in air-filled voids on the cave-ceiling, in the epikarst, and overlying soil (Johnson et al., 2006). Air-filled voids have reduced pCO_2 in comparison to infiltrating water, which enhances CO_2 degassing and the precipitation of calcite (Borsato et al., 2016). Enhanced CO_2 degassing leads to the preferential loss of ^{12}C in the percolating waters, increasing the $\delta^{13}C$ values in the remaining solution and speleothem. Additionally, when calcite precipitates it preferentially uptakes Ca^{2+} leaving the remaining solution and speleothem enriched in the divalent ions (Mg^{2+}, Sr^{2+}, Ba^{2+}). Therefore, we interpret the covariation in $\delta^{13}C$ and Mg/Ca in our record to be reflective of prior calcite precipitation, primarily controlled by local water balance, not necessary precipitation amount. It is important to note during the transition out of the Pleistocene the increasing trend in Mg/Ca is anomalous compared to $\delta^{13}C$ ($r = 0.14$, $p = 0.04$) and $\delta^{18}O$, suggesting trace elements do not match our other proxies on orbital timescales, contributing to the weak correlation between Mg/Ca and $\delta^{13}C$ over the entire record ($r = 0.22$, $p < 0.01$).

Supplementary Note 5

Mg/Ca values diverge from the stable isotope record during the glacial-interglacial transition, with an increase from mean glacial concentrations of 30 mmol/mol to Holocene concentrations of ~40 mmol/mol (Fig. 2). The increase in Mg/Ca ratios could be reflective of dryer conditions during the Holocene, however, we interpret the rise in Mg/Ca ratios due to non-hydrologic controls on speleothem trace element ratios. During the late-Pleistocene, we find an overall agreement between Mg/Ca and $\delta^{13}C$, indicative that hydrologically sensitive PCP is likely an important controlling mechanism. The divergence between these proxies during the deglacial and Holocene, however, may be explained by the competing influence of temperatures on Mg partitioning into calcite. Cave temperatures are reflective of mean annual surface temperatures (Dorale, 1998), therefore as temperatures increased from the glacial through mid-Holocene the temperature dependent partition coefficient for Mg was likely affected (Stoll et al., 2012). If we assume a 5°C interglacial-glacial temperature correction for Mg/Ca, modeling studies demonstrate this change could impart up to a 23% increase in Mg/Ca (Stoll et al., 2012), consistent with the observed shift in CB2.

Importantly, the influence of temperature on Mg/Ca ratios during Heinrich Stadials throughout the late-Pleistocene is much less prevalent. If temperature strongly modulated Mg/Ca on these timescales, it would theoretically lead to decreased Mg/Ca ratios in response to cool Heinrich Stadials. However, Mg/Ca ratios generally increase during these millennial scale events, suggesting PCP is the more dominant controlling mechanism. This is further supported by the moderate to strong correlation of raw, unsmoothed, Mg/Ca ratios to $\delta^{13}C$ during key paleoclimate events including HS2 (23-26 ka, $r = 0.71$, $p = 0.01$), HS3 (28 – 32 ka, $r = 0.77$, $p < 0.01$), HS4 (40-43 ka, $r = 0.54$, $p < 0.01$), HS5 (47-50 ka, $r = 0.51$, $p < 0.01$), and HS6 (58-62 ka,

r = 0.55, p < 0.01). Correlation values presented in this paper were generated using BINCOR, an R-studio statistical (Polanco-Martinez et al., 2019). Correlations were calculated using the *bin_cor* function which calculates the correlation between two uneven time series and incorporates the memory of the data using an AR1 model. A FLAGTAU of 3 was selected, which utilizes Monte-Carlo simulations to estimate bin-width and calculate the number of bins.

Supplementary Note 6

Precipitation samples collected from June 2018 to May 2019 fall closely to the Global Meteoric Water Line with $\delta^{18}O$ values ranging from -14.03‰ to 0.22‰ and δD values ranging from -98.3‰ to 6.7‰ (VSMOW) (Fig. S2). In total 48 samples were collected and the amount-weighted mean $\delta^{18}O_{precip}$ is -4.84 ± 2.81‰. Monthly-averaged precipitation amount from a nearby weather station and $\delta^{18}O$ values demonstrate a strong correlation ($r^2 = 0.88$) (Fig. S2), suggesting that precipitation $\delta^{18}O$ is reflective of precipitation amount on seasonal timescales. However, these results do not directly confirm the dominance of the amount effect on interannual or longer timescales, and we cannot rule out additional influence of other factors, such as moisture source region, which could enhance or diminish this signal. HYSPLIT analyses, however, demonstrate that the moisture at our field site is dominantly sourced from the Gulf of Mexico and Caribbean Sea (Fig. S1). Even during boreal winter, the Pacific contributes a relatively insignificant amount of moisture (< 5 g/kg m/s). Paleoclimate model simulations (MIROC-ESM, CNRM-CM5, MPI-ESM-P, GISS-E2-R) conducted as part of the Paleoclimate Model Intercomparison Project 3 (PMIP3) (Braconnot et al., 2012) do not demonstrate any major re-organization of wind patterns or changes in seasonality of precipitation (JJAS still dominant) during the mid-Holocene and Last Glacial Maximum (Fig. S9, Fig. S10). To investigate interannual $\delta^{18}O$ variability, we analyzed results from the reanalysis nudged isotope enabled IsoGSM model (Yoshimura et al., 2008) for our study region. A spatial correlation between annual precipitation $\delta^{18}O$ from IsoGSM with GPCC v.7 precipitation (Fig. S2) shows a clear negative correlation over NE Mexico, further supporting interpretation of $\delta^{18}O$ primarily as a rainfall amount signal, and supporting previous work from nearby Xalapa (Goldsmith et al., 2012). Therefore, we conclude that precipitation, and hence stalagmite, $\delta^{18}O$ at our site is likely reflective of local and regional precipitation amount rather than changes in moisture source.

Supplementary Note 7

Drip water δD and $\delta^{18}O$ samples from Cueva Bonita plot relatively closely to local precipitation samples, with the mean $\delta^{18}O_{drip}$ of -4.27 ± 0.87‰ well within the standard deviation of the amount-weighted mean precipitation of -4.84 ± 2.81‰ (Wright et al., 2022). This suggests mean cave drip water $\delta^{18}O$ could retain the majority of the $\delta^{18}O$ signal from overlying precipitation, but more samples are needed to confirm. However, the data we've collected thus far suggests drip water samples from Cueva Bonita are overall reflective of annual overlying precipitation $\delta^{18}O$, and therefore reflective of precipitation amount on annual timescales. The small 0.57‰ observed difference between precipitation and drip water $\delta^{18}O$ could be attributed to evaporation,

analytical uncertainty, groundwater mixing or soil chemistry, but the slope of drip water $\delta^{18}O$ and $\delta^{2}D$ suggests evaporation is the most likely culprit. Evaporation is evident with a shift in slope from 7.9 in the local meteoric water line (LMWL) to 6.1 in the trendline of the drip waters. However, the deviation from the LMWL is minor, could be driven by a single data point, and does not inhibit the use of precipitation $\delta^{18}O$ to reconstruct past changes in relative precipitation amount (wetter/drier) assuming calcite is deposited near isotopic equilibrium.

Supplementary Note 8

Modern glass-plate calcite from Cueva Bonita, which grew from 2018 to 2019, has a $\delta^{18}O$ value of -4.78 ± 0.08‰. Utilizing the mean (n = 2) $\delta^{18}O_{drip}$ from 2018 and 2019 (-4.84 ± 0.93‰) and the average drip interval (11s), cave air pCO_2 (800 ppm), water pCO_2 (16,000 ppm), temperature (17.3°C), relative humidity (100%), and ventilation (0 m/s), the predicted $\delta^{18}O$ of calcite deposited in isotopic equilibrium is -4.77‰, very close to the measured value. Cave monitoring demonstrates seasonal to interannual variability in parameters known to drive disequilibrium isotope fractionation. For instance, the drip interval can be highly variable and there is a ~900 ppm change in seasonal cave air pCO_2 (Fig. S3) which could cause rapid CO_2 degassing, further increasing fractionation. We therefore utilized a geochemical proxy system model (Deininger & Scholz, 2019) to quantitatively evaluate the impact of the individual and combined influences of these parameters on speleothem isotope chemistry at Cueva Bonita.

Supplementary Note 9

Surprisingly, model results suggest the influence of relative humidity on speleothem $\delta^{18}O$ is very minor (Fig. S5). Even at 0% relative humidity there is only a 0.05% change in $\delta^{18}O$ at the longest drip interval (100s). Changes in the drip interval appear to drive a more noticeable, but still relatively small, change in $\delta^{18}O$ with up to 0.25‰ at 100% relative humidity. While previous studies have shown relative humidity (RH) can drive a large magnitude shifts in $\delta^{18}O$ (Deininger et al., 2012; Deininger & Scholz, 2019; Mühlinghaus et al., 2009), results presented here, which include changes in RH considerably larger than those observed at any drip site at Cueva Bonita, suggest the oxygen isotopic composition of speleothems at this cave are not particularly sensitive to changes in relative humidity. We suggest this insensitivity is driven by the sufficient replenishment of water on the speleothem surface (low drip interval), inhibiting significant evaporation. Lastly, changes in cave air pCO_2 could also alter the oxygen isotope composition during deposition observed to vary seasonally. However, Figure S5 demonstrates cave air pCO_2 drives less than a 0.1‰ change in speleothem $\delta^{18}O$ at a 100s drip interval. Cumulatively, model results demonstrate that cave variability does not have a significant impact on speleothem $\delta^{18}O$, reinforcing the interpretation that Cueva Bonita calcite is deposited very close to isotopic equilibrium and is reflective of cave temperature and precipitation $\delta^{18}O$.

Supplementary Note 10

The ultimate moisture source of any precipitation is seawater. In order to correct precipitation for glacial-interglacial shifts in $\delta^{18}O_{sw}$, we subtracted interpolated $\delta^{18}O_{sw}$ values (Waelbroeck et al., 2002) from CB2 data (Fig. S15). This ultimately decreases glacial-interglacial $\delta^{18}O_{speleothem}$ values but does not significantly impact the amplitude of millennial scale variability.

Supplementary Note 11

Calcite samples were analyzed for ^{14}C at University of California, Irvine within the Keck Carbon Cycle Accelerator Mass Spectrometry (KCCAMS) laboratory. Calcite powders were leached with 10% HCl acid, to remove any secondary carbonates, and hydrolyzed with 85% phosphoric acid. Using a modified hydrogen-reduction method (Beverly et al., 2010), samples were then graphitized onto a Fe catalyst. During data processing calcite powder from a radiocarbon free speleothem was used for blank subtraction.

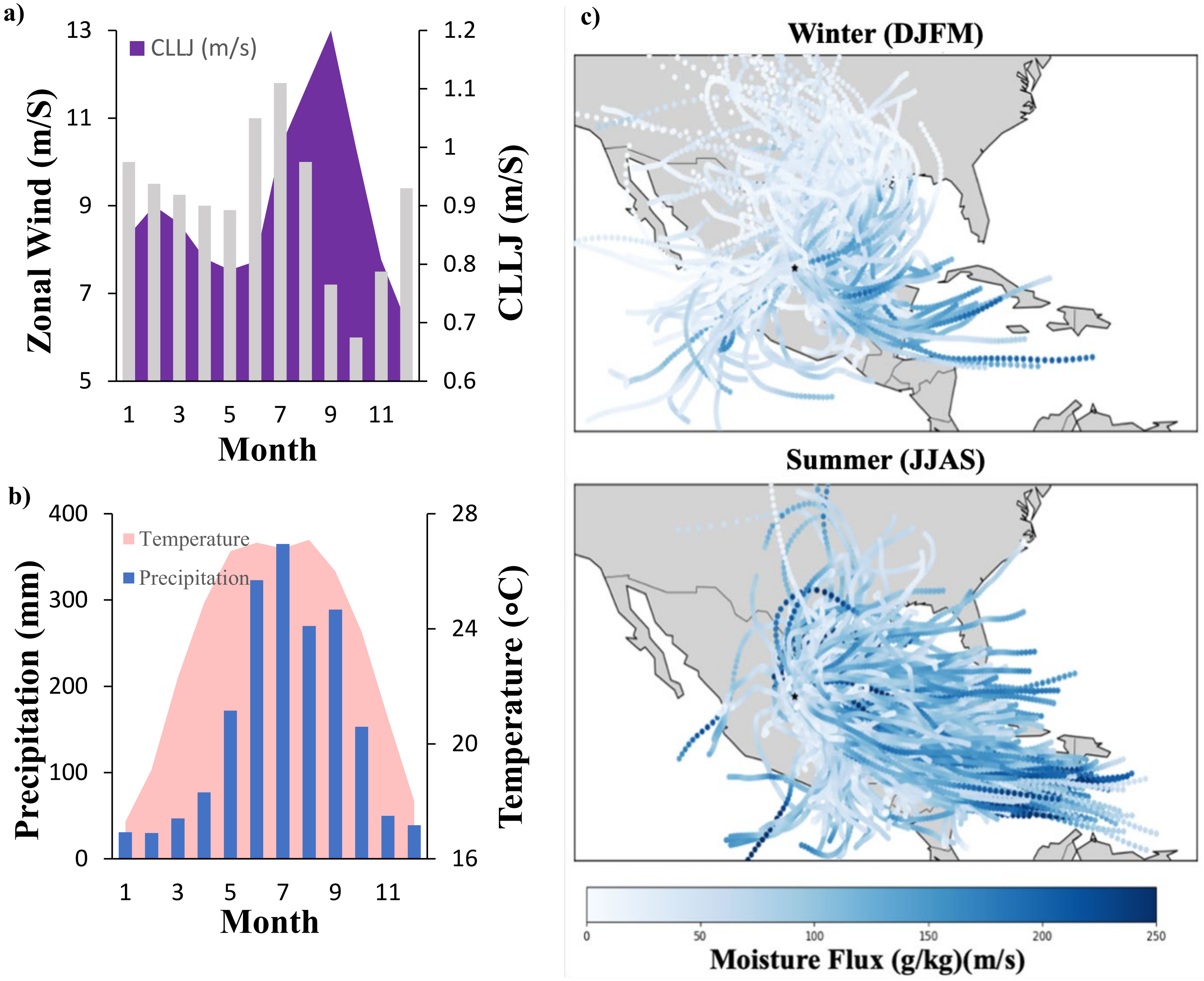

Fig. S1. Modern precipitation patterns of NE Mexico. a) Monthly average precipitation and temperature above cave site from www.weatherbase.com. **b)** Monthly average zonal winds in the Caribbean Basin and strength of Caribbean Low-Level Jet (Mestas-Nuñez et al., 2007). **c)** Moisture flux trajectory analysis conducted with NOAA HYSPLIT model (Stein et al., 2015) in conjunction with the python package PYSPLIT (Cross, 2015) for increased computational efficiency. Back-trajectory air trajectories yielding moisture at Cueva Bonita (n=3600) demonstrate the dominant moisture source is from the Gulf of Mexico and Caribbean Sea during both boreal winter and summer.

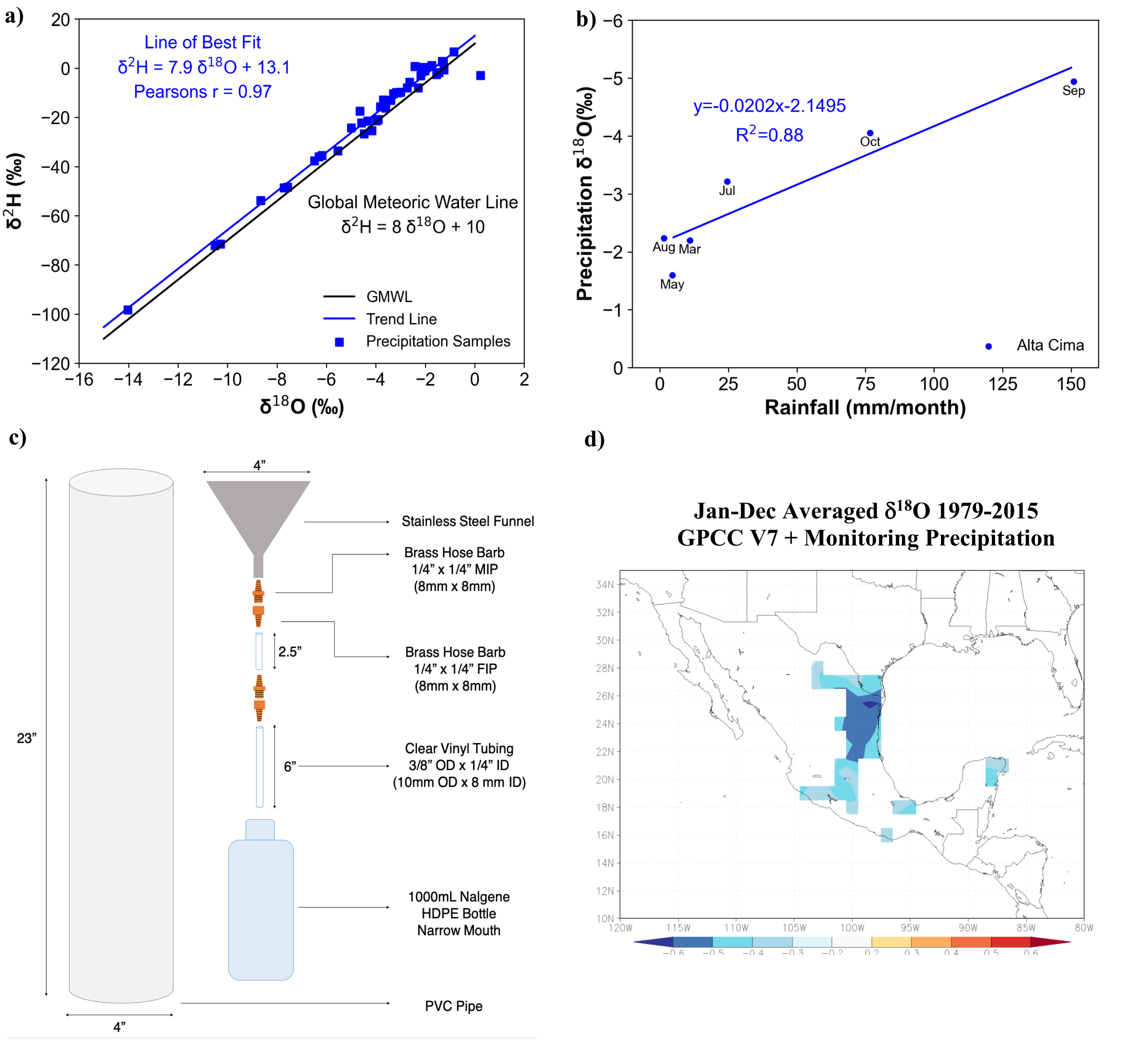

Fig. S2. Isotopic composition of precipitation. a) Precipitation $\delta^{18}O$ and $\delta^{2}H$ for 48 precipitation samples collected above the cave near the city of Alta Cima. Almost all samples fall on the GMWL. **b)** Monthly averaged precipitation from a nearby weather station (Gómez-Farías, 6km from Cueva Bonita, source:www.weatherbase.com) with precipitation $\delta^{18}O$ collected above the cave. **c)** Design of precipitation collectors built to limit wind flow and atmospheric interaction. This system relies on a series of barbs and tubing to provide a more cost-effective alternative to mineral-oil precipitation collectors. All parts were assembled and glued together using hot glue and/or gorilla glue. In a 7-day trial where collectors were left in the sun in 80°F weather, no evaporation was detected. **d)** Spatial correlation of annual weighted mean precipitation $\delta^{18}O$ (1979-2015) from IsoGSM gridpoint nearest Cueva Bonita with annual GPCC v7 precipitation (1979-2015), illustrating a negative correlation between precipitation oxygen isotope ratios and regional precipitation amount in our study area.

a)

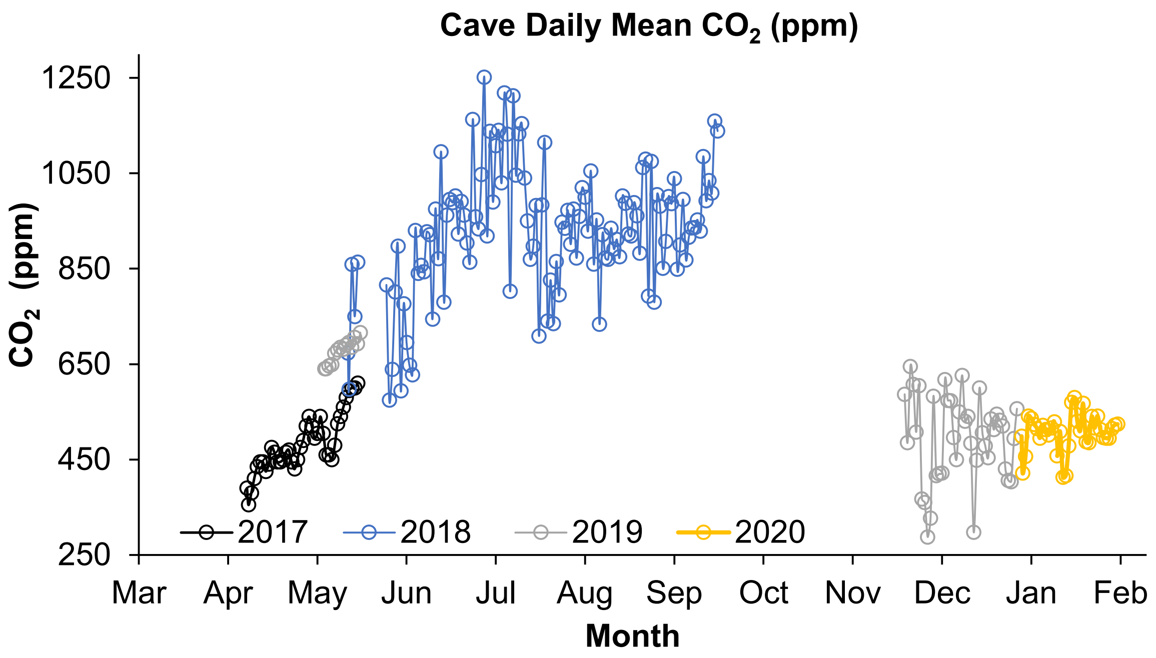

b)

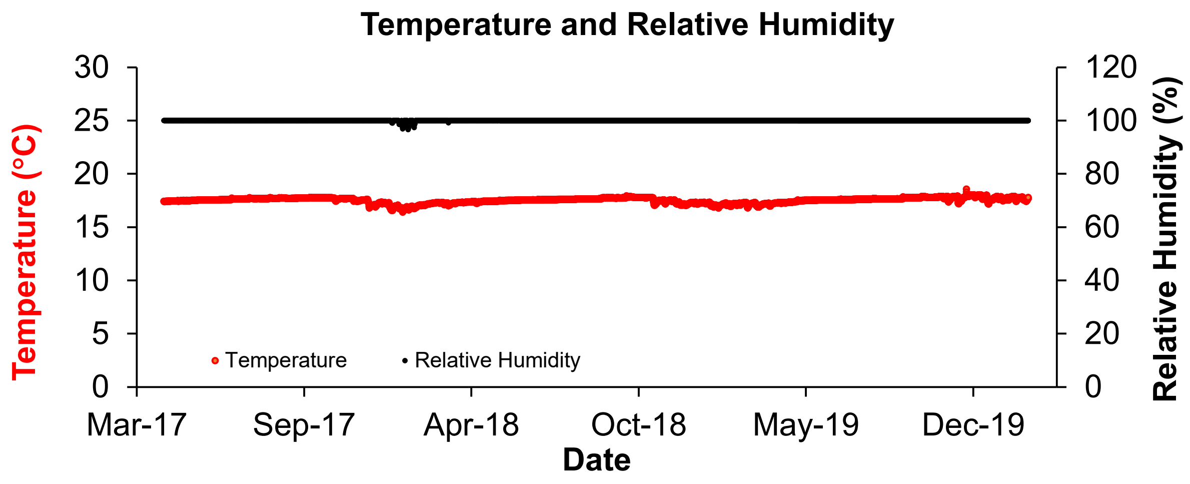

Fig. S3. Cave temperature, relative humidity from 2017-2020. a) Daily mean CO_2 for Cueva Bonita recorded every 3-12 hours using a hand-held Vaisala GM-70 CO_2 monitor attached to a 12 Volt lantern battery. Higher pCO_2 levels are noted during summer, compared to all other seasons, with a maximum interannual fluctuation of ~900 ppm. We place more emphasize on the magnitude and temporal variability of pCO_2 due to lower than atmospheric concentrations during the winter and spring, possibly caused by poor instrumental calibration. Colors indicate various years of data. While Co2 monitors were calibrated before use, we acknowledge some measurements are below atmospheric levels **b)** Temperature and relative humidity in Cueva Bonita recorded from April 2017 until February 2020. Measurements were made every 2 hours using HOBO loggers. Temperature remains relatively constant at 17 and relative humidity at 100%.

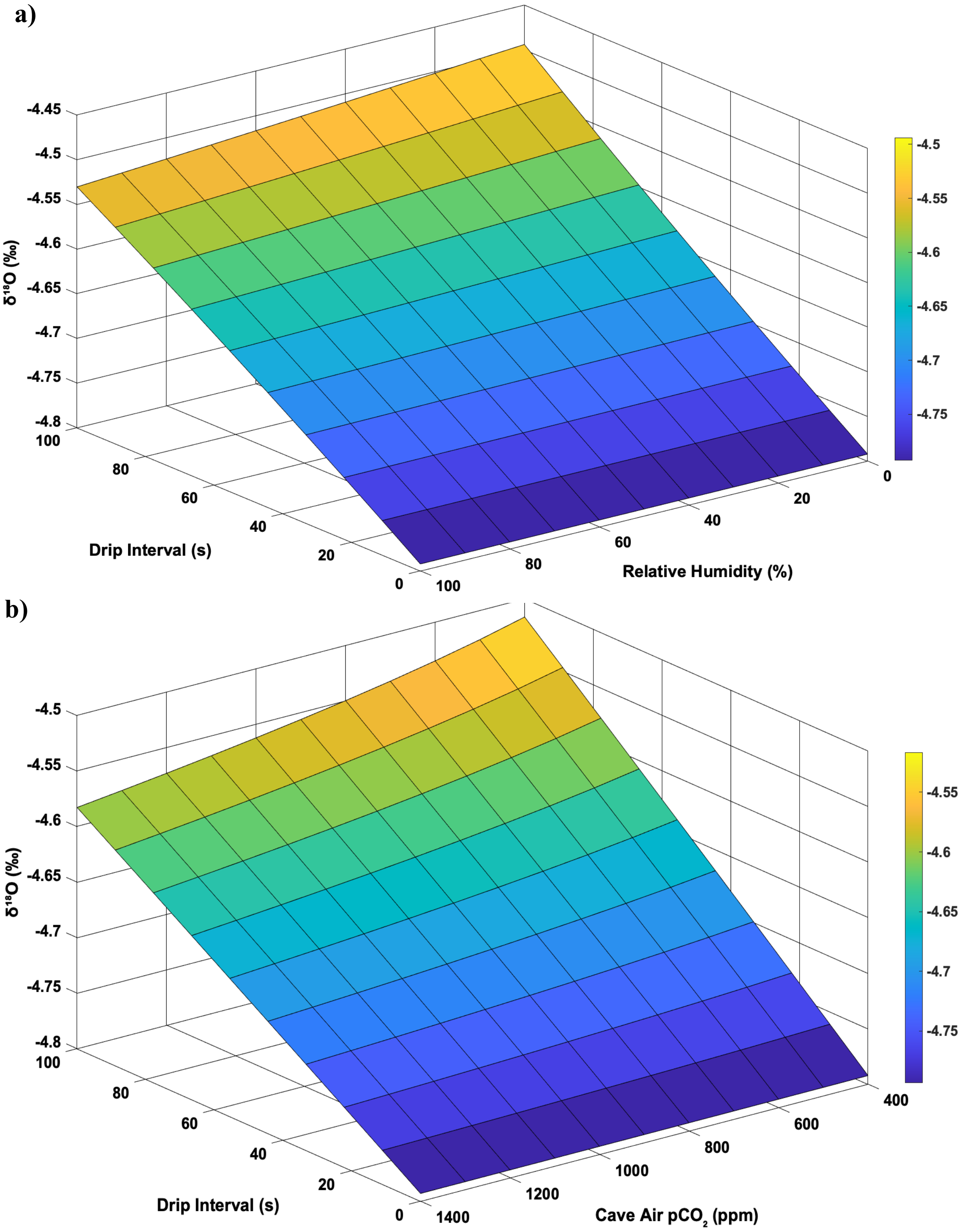

Fig. S4. Modelled results of modern calcite δ^{18}O to cave variability. a) Isolution results which demonstrates the response of calcite δ^{18}O to changes in relative humidity and drip interval. Variation in drip intervals (seconds between drips) and relative humidity were overexaggerated compared to real world measurements, but still only contributed a ~0.3‰ change in calcite δ^{18}O. **b)** Demonstrates the response of δ^{18}O to changes in cave air pCO_2 and drip interval. Low pCO_2 and high drip intervals only contribute a ~0.3‰ change in calcite δ^{18}O.

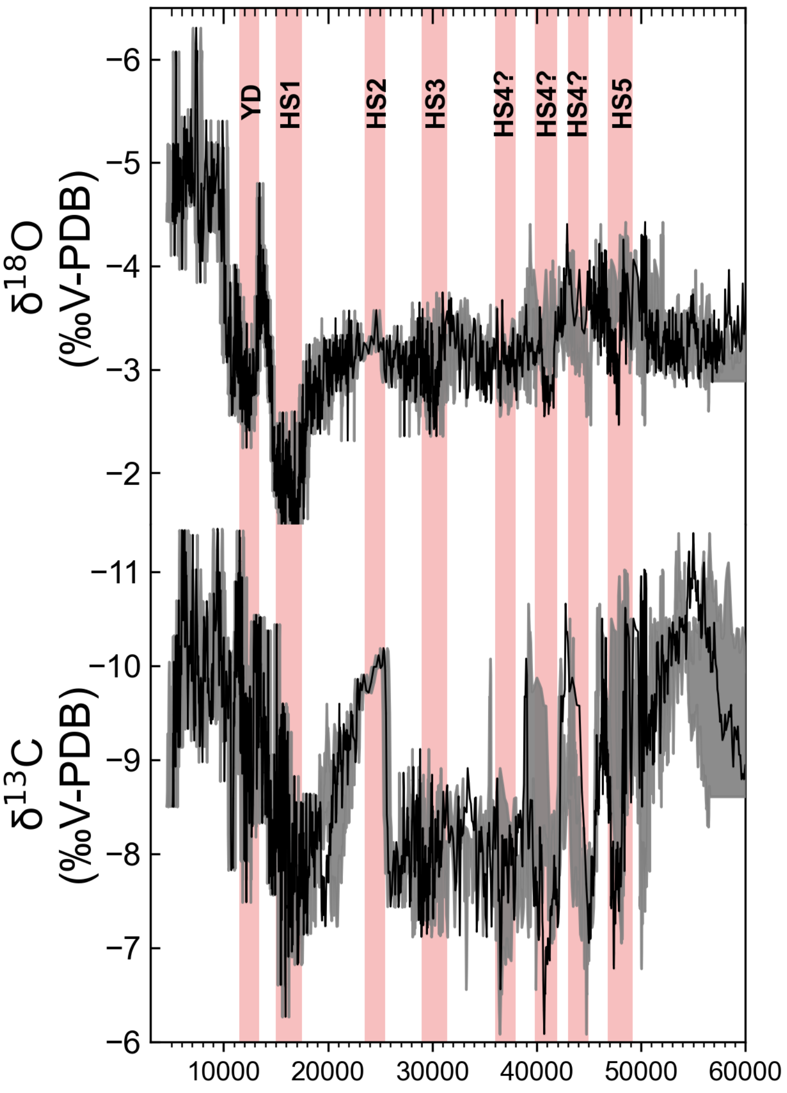

Fig. S5. CB2 $\delta^{18}O$ and $\delta^{13}C$ with age uncertainties. CB2 $\delta^{18}O$ and $\delta^{13}C$ with age uncertainties demonstrate HS4 could have occurred as early as 38-36 ka or as late as 45-43 ka in the CB2 record. The 95% confidence interval (black) suggests HS4 occurred between 42-40 ka.

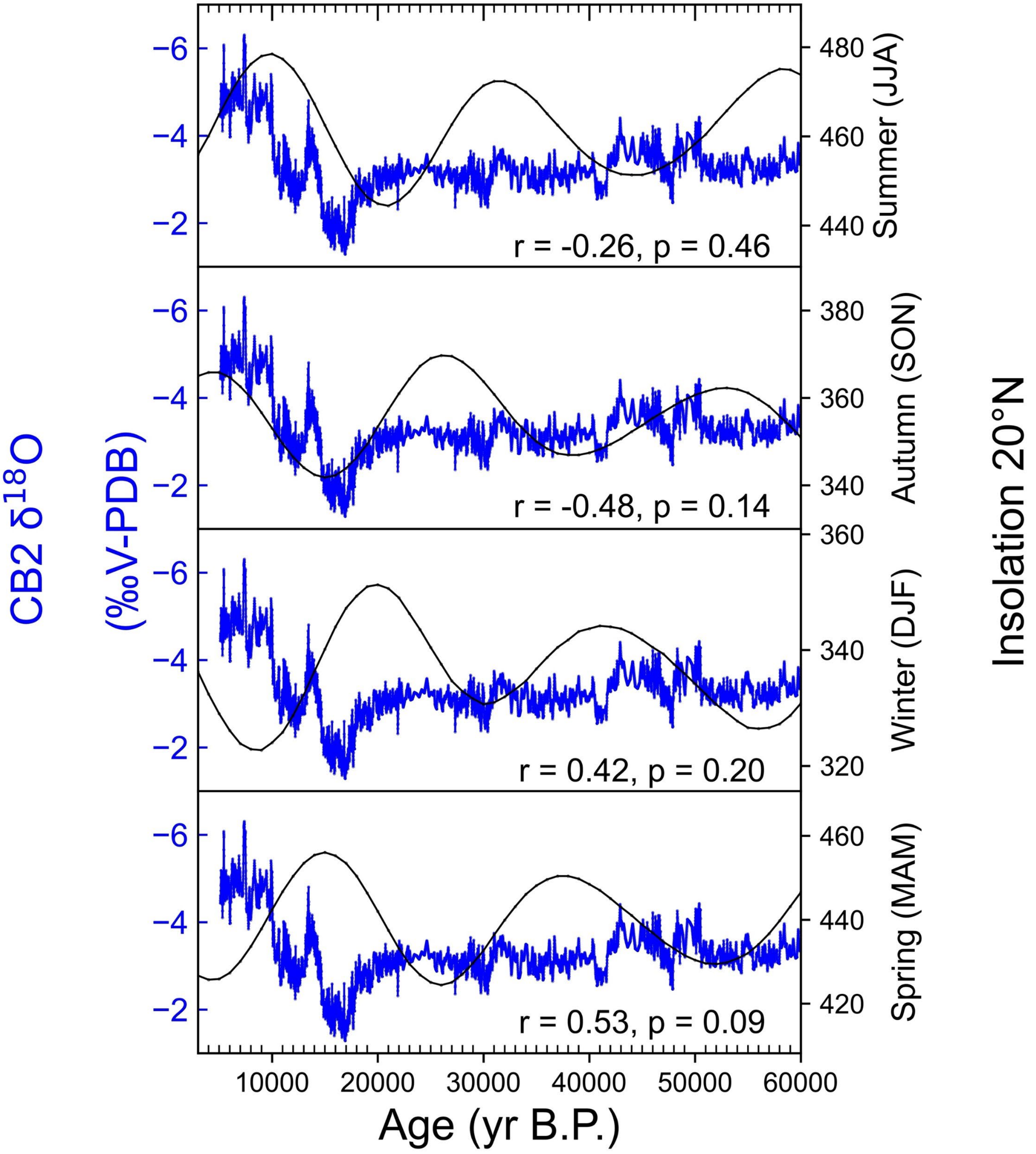

Fig. S6. CB2 $\delta^{18}O$ (blue) compared to various seasons of insolation. While autumn and summer insolation appear to have a strong negative correlation to CB2 $\delta^{18}O$ (positive correlation to rainfall) over the Pleistocene – Holocene transition, this co-variation does not continue over the late Pleistocene. We also do not see a significant correlation to Winter (DJF) or Spring (MAM) insolation.

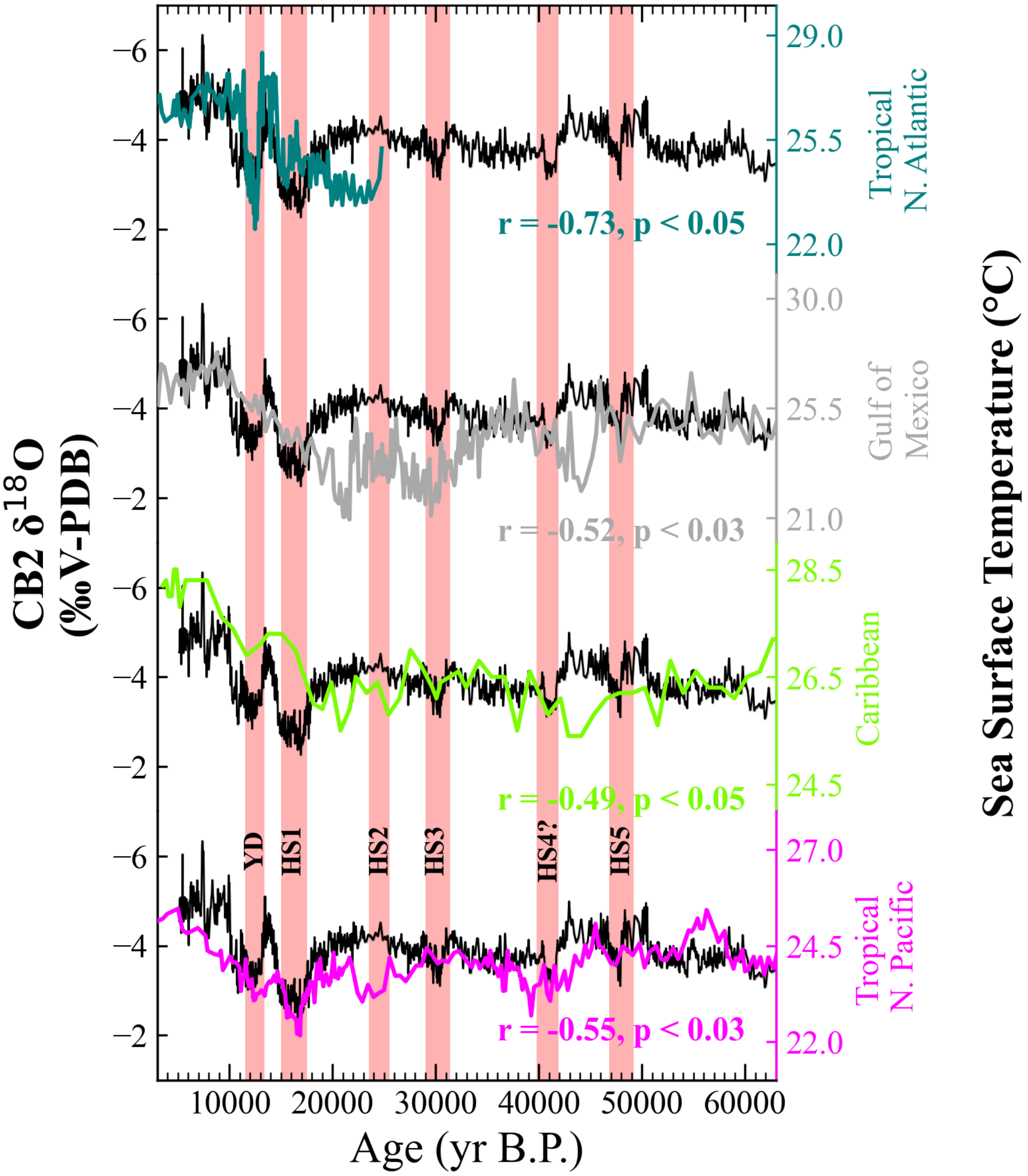

Fig. S7. CB2 δ^{18}O (black) compared to regional SSTs. CB2 is plotted with Gulf of Mexico (r = -0.52, p < 0.03, silver, Ziegler et al., 2008), Caribbean (r = -0.59, p < 0.05, lime green, Schmidt et al., 2004), Tropical N. Pacific (r = -0.55, p < 0.03, magenta, Dubois et al., 2011) and Tropical N. Atlantic SSTs (r = -0.73, p < 0.01, teal, Lea et al., 2003).

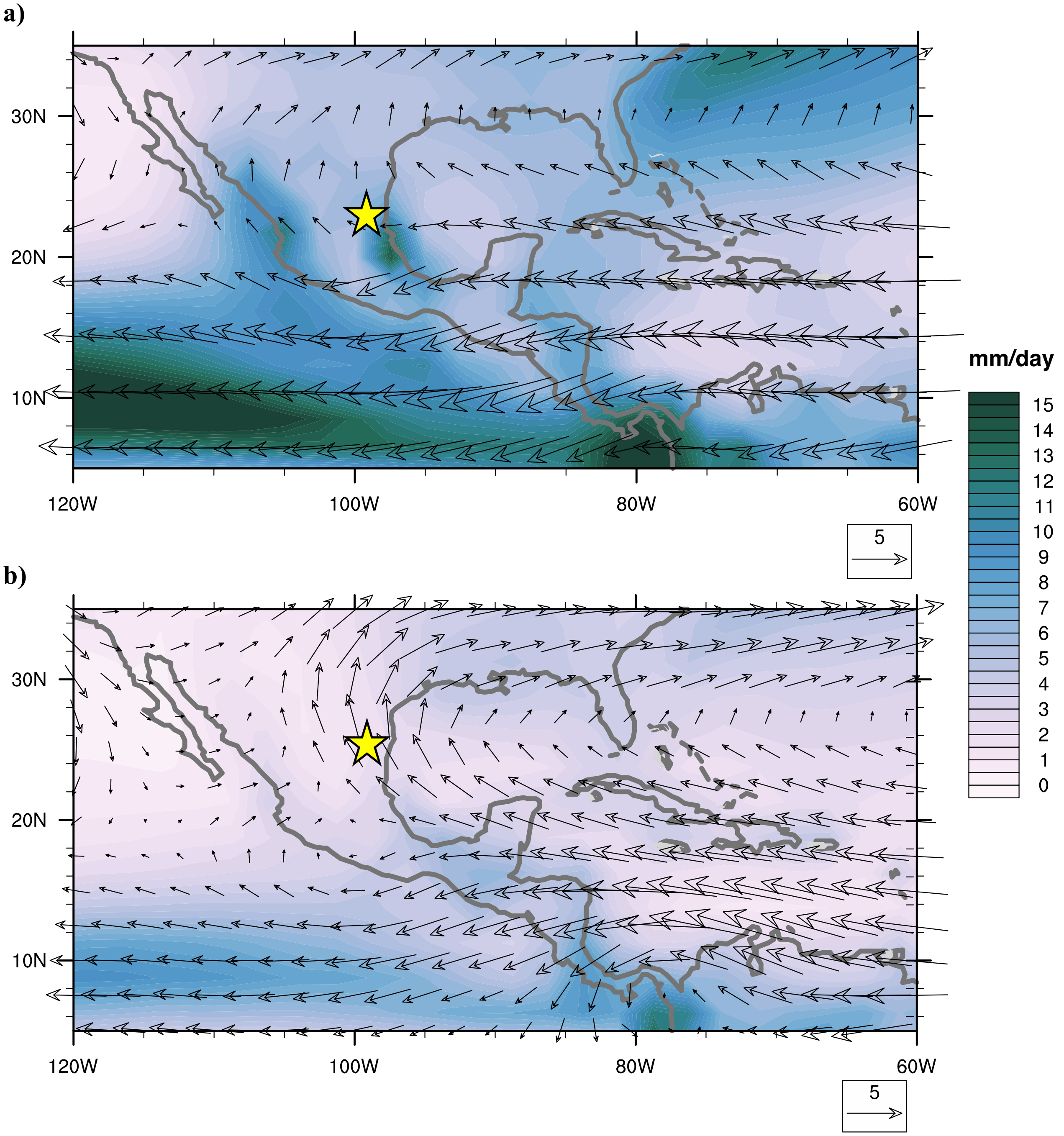

Fig. S8. iCESM1 precipitation and rainfall data compared to observations. a) iCESM results of pre-industrial low-level winds and precipitation patterns. **b)** Merged observational rainfall data from the Global Precipitation Climatology Project (GPCP; Adler et al., 2018) and low-level winds from NCEP-NCAR reanalysis.

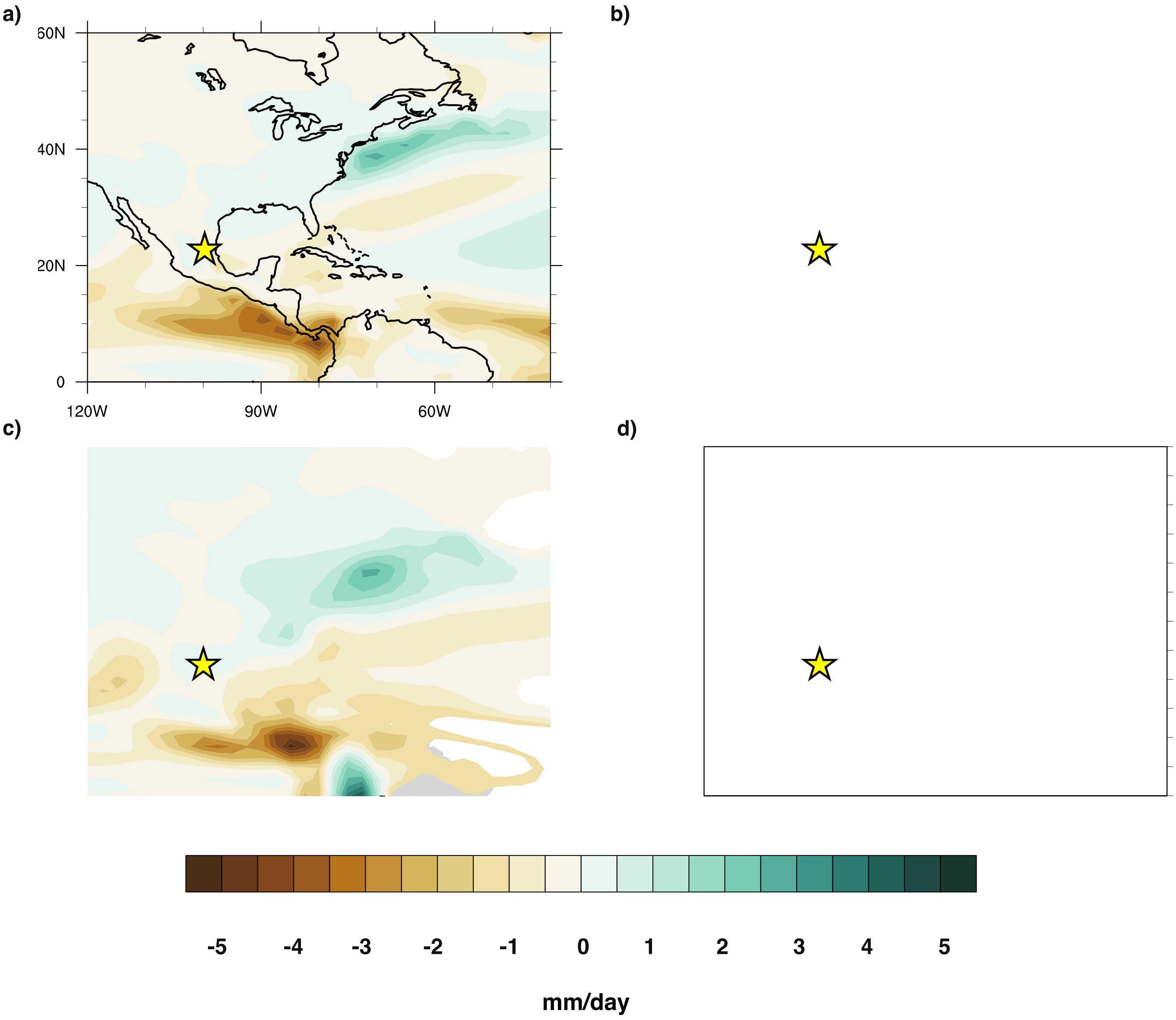

Fig. S9. Moisture budget analysis of precipitation change. (A) Net precipitation change (P-E). **(B)** Thermodynamic influence on P-E. **(C)** Dynamic influence on P-E. **(D)** Transients/higher resolution term influence on P-E.

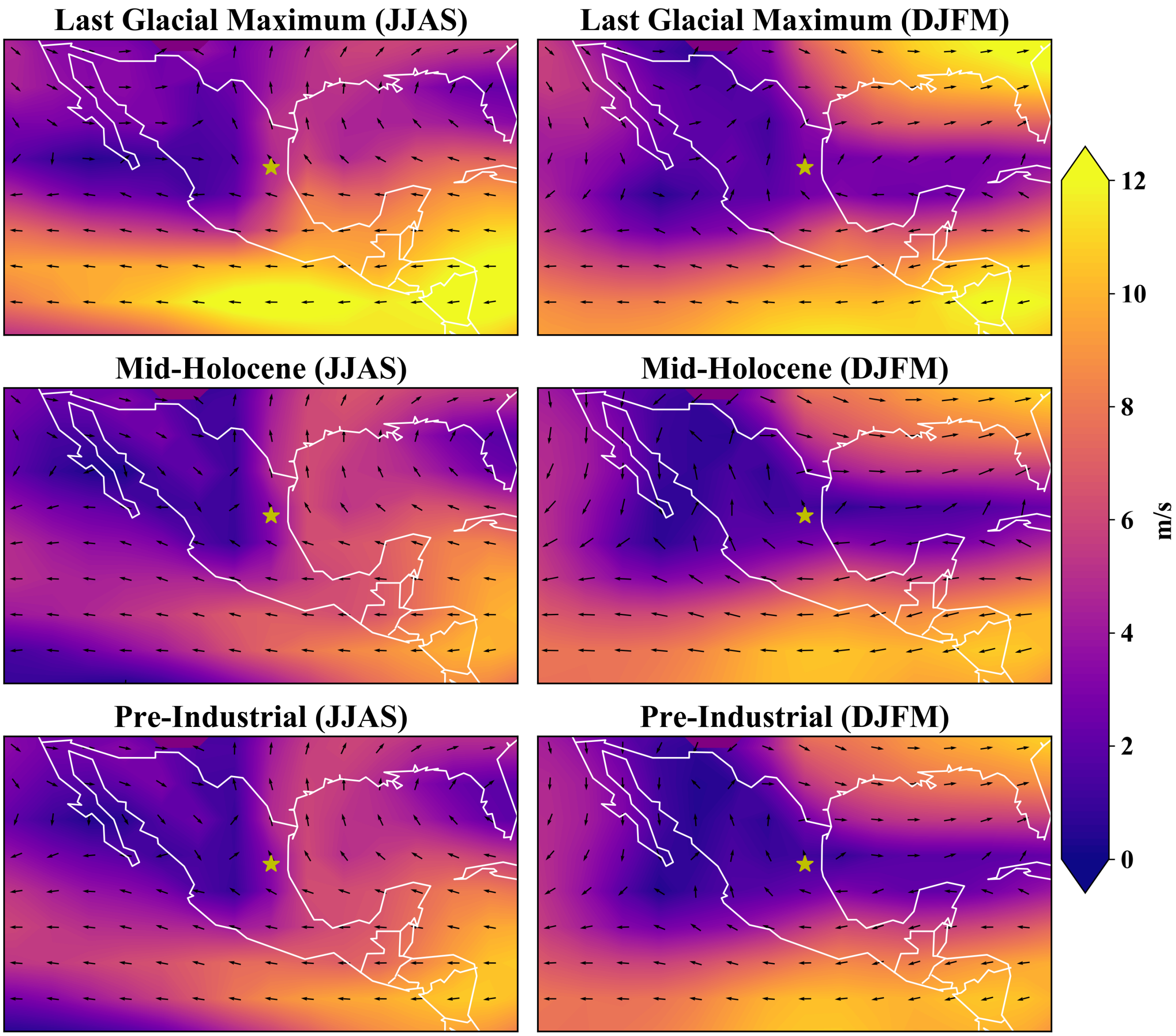

Fig. S10. Summer and winter low level winds during the Last Glacial Maximum, Mid-Holocene and Pre-Industrial Period. Summer (left) and winter (right) low-level wind direction and magnitude during the LGM (top), Mid-Holocene (mid) and Pre-Industrial (bottom) time periods from the NASA model GISS-E2-R (Braconnot et al., 2012). The magnitude is illustrated by color, direction is indicated by arrow. Winds were noticeably stronger during the Last Glacial Maximum (top), indicated by yellow, however, there is no major changes in in wind direction with respect to the Mid-Holocene or Pre-Industrial period that would suggest an increased proportion of rainfall from the Pacific.

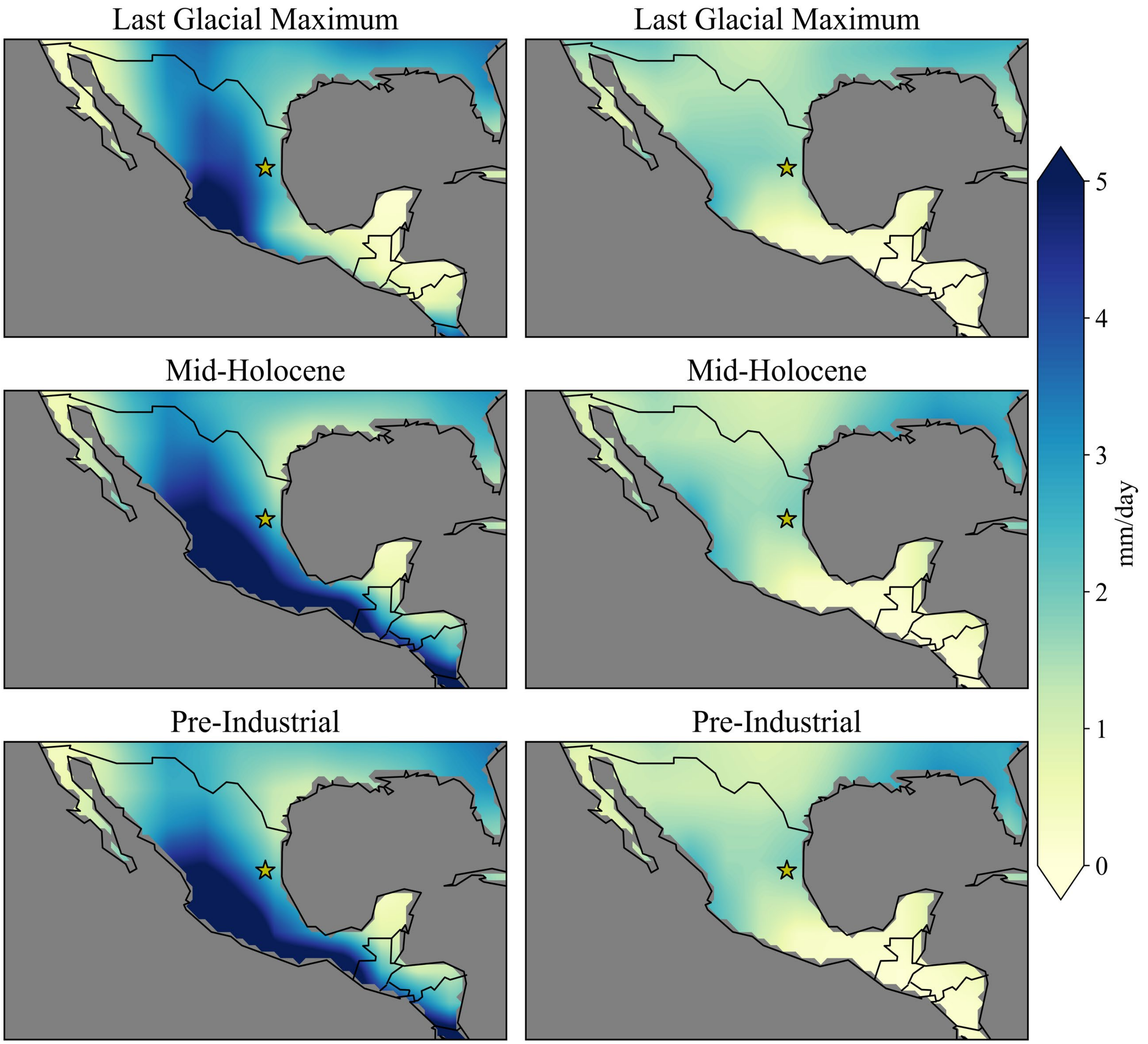

Fig. S11. Summer and winter precipitation during the Last Glacial Maximum, Mid-Holocene and Pre-Industrial Period. Precipitation results from PMIP3 Model GISS-E2-R (Braconnot et al., 2012). Summer (left) and winter (right) precipitation during the LGM (top), Mid-Holocene (mid) and Pre-Industrial (bottom) time periods. The model demonstrates increased precipitation throughout time (LGM<MI<PI), however, summer remains the wet season throughout all time periods.

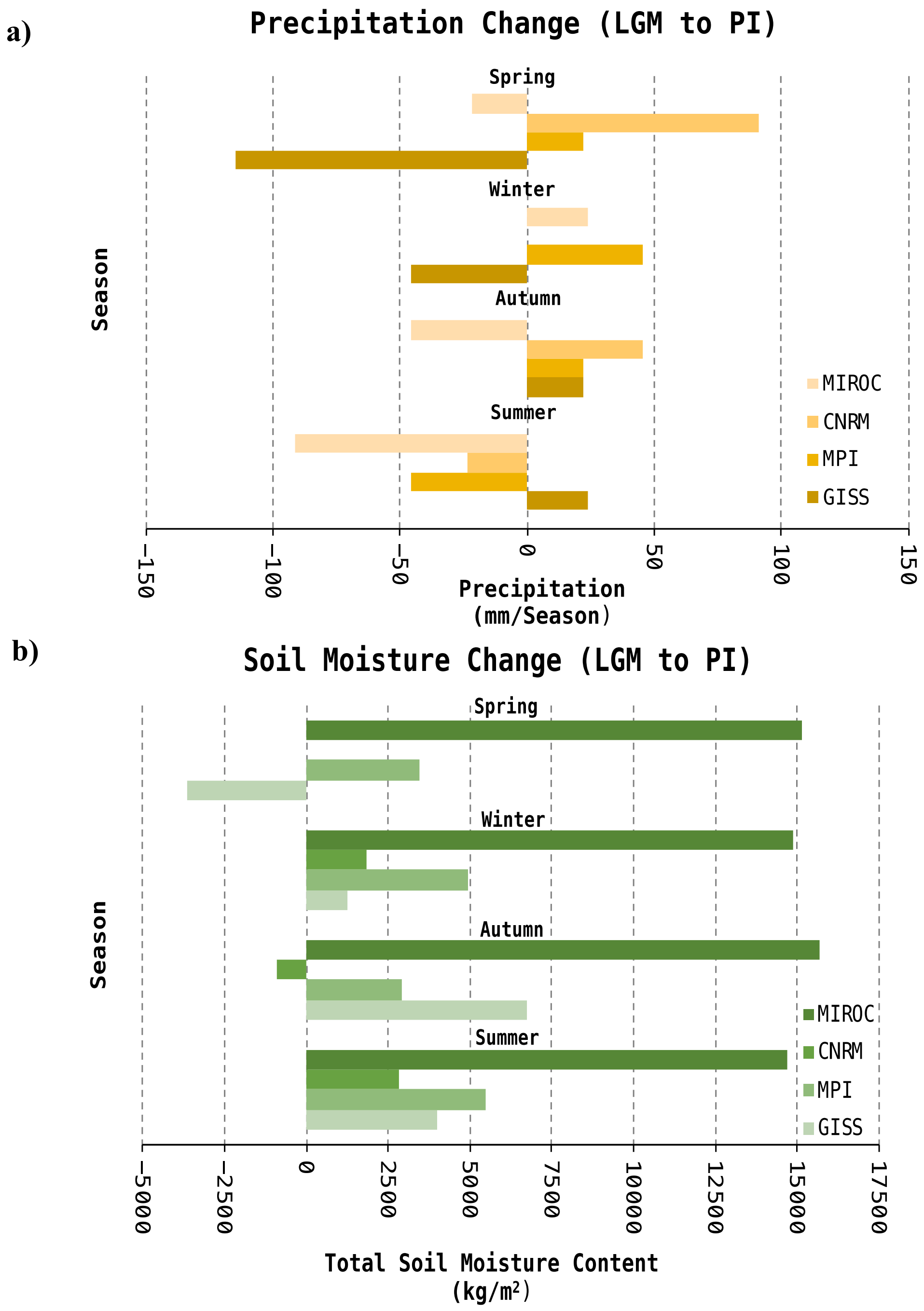

Fig. S12. Precipitation and soil-moisture comparison between the Pre-Industrial to Last Glacial Maximum. a) Comparison of LGM precipitation and **b)** soil moisture during the LGM and Pre-industrial for 4 of the best performing PMIP3 models during Spring (MAM), Winter (DJF), Autumn (SON) and Summer (JJA) averaged over 22°N-24°N and 98°W-100°W. It is unclear if precipitation increased or decreased during the LGM compared to PI. However, all models show increases in total soil moisture, suggestive of decreased evaporation during the LGM. The four PMIP3 models utilized in this analsyis (CNRM, GISS, MPI and MIROC) were chosen because they have been shown to accurately reproduce Last Glacial Maximum precipitation in a proxy-model comparison study of the African Monsoon (Chevalier et al., 2017).

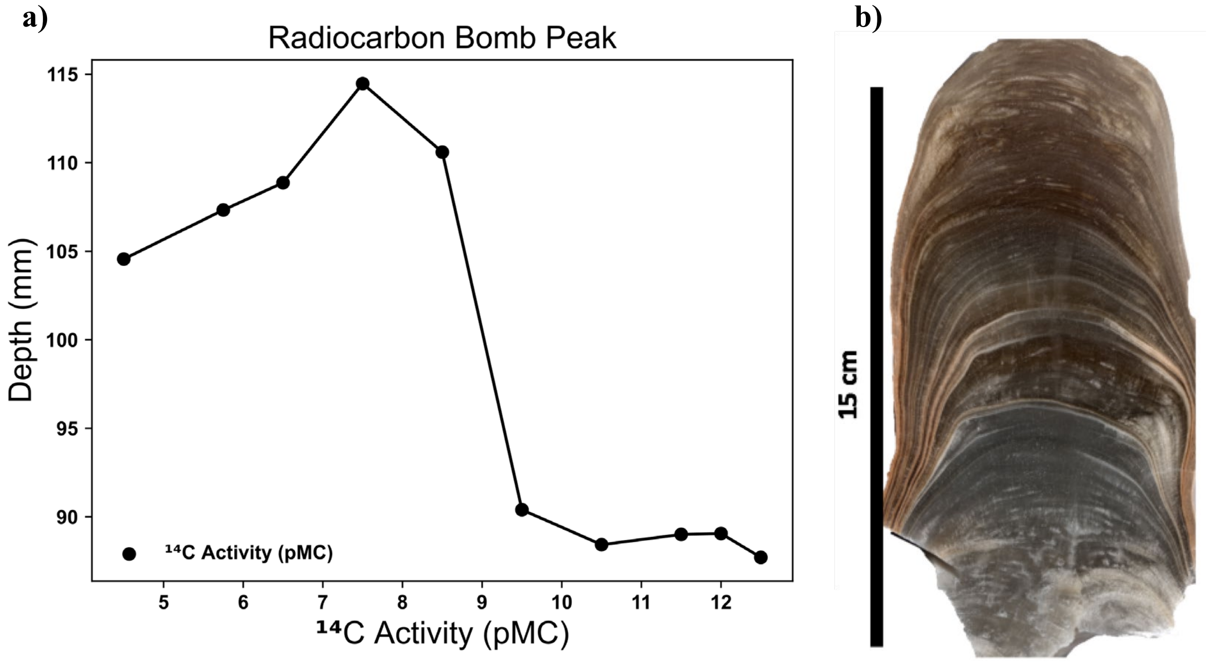

Fig. S13. Radiocarbon analysis of a modern speleothem. a) Speleothem ^{14}C activity in the top 12 mm of sample CB4, a modern speleothem sample also collected from Cueva Bonita. The rise of the bomb peak starts around a depth of ~10 mm and should not lag more than ~1 - 2 years behind the start of the rise of atmospheric bomb peak in 1955 (Genty and Massault 1999; Hodge et al., 2011). **b)** Speleothem sample CB4 also collected from Cueva Bonita.

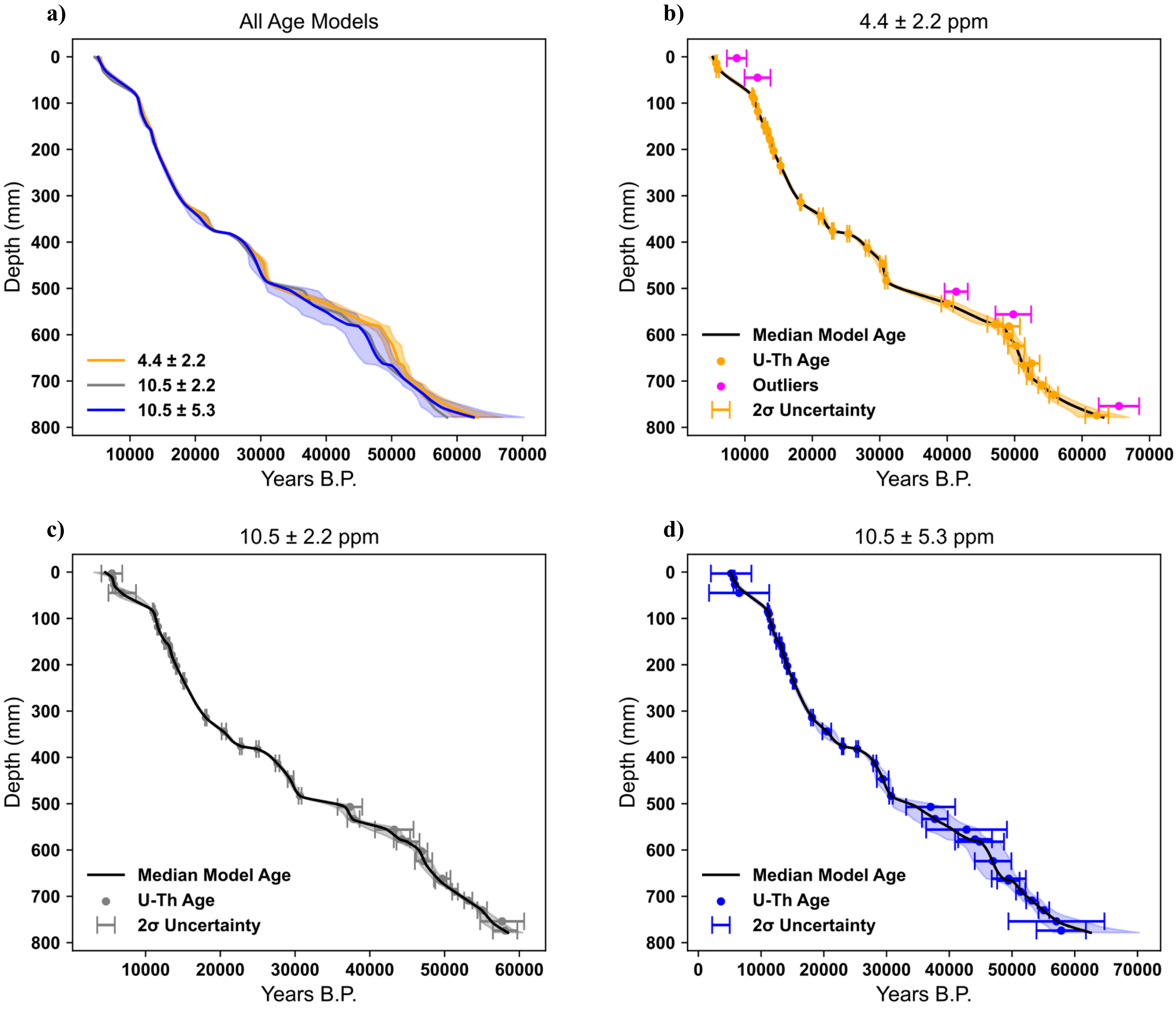

Fig. S14. CB2 age model with various initial $^{230}Th/^{232}Th$ values and uncertainties. a) All age models constructed using COPRA show strong similarities despite different initial Th corrections **b)** CB2 age model constructed using the standard correction, with an initial $^{230}Th/^{232}Th$ value of 4.4 ± 2.2 ppm **c)** CB2 age model constructed using the stratigraphically determined correction without a corresponding increase in uncertainty (10.5 ± 2.2 ppm). **d)** CB2 age model constructed using the stratigraphically determined correction with a scaled 50% uncertainty (10.5 ± 5.3 ppm).

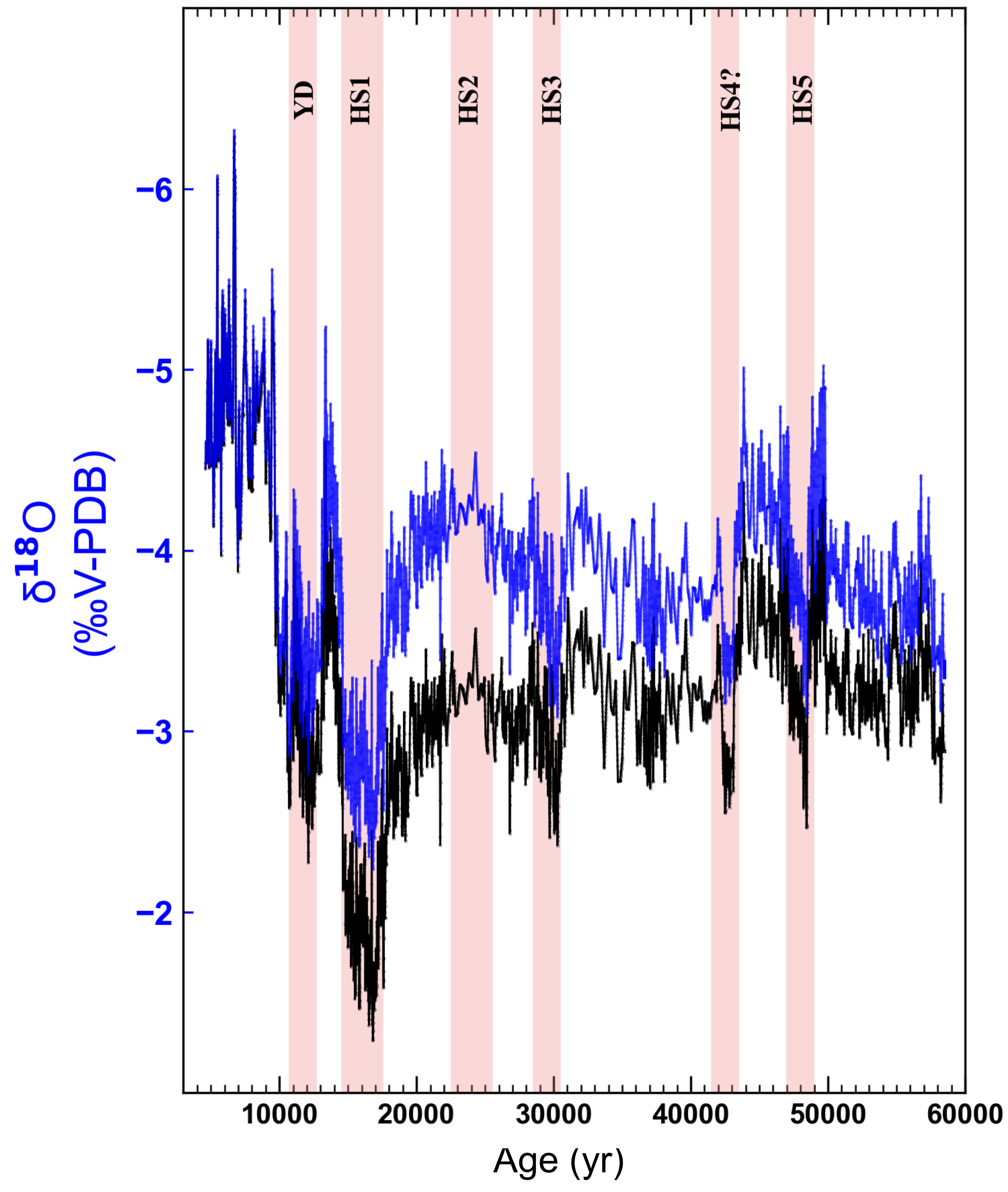

Fig. S15. Comparison of raw CB2 $\delta^{18}O$ with mean-seawater subtracted CB2 $\delta^{18}O$. Mean seawater interglacial-glacial $\delta^{18}O$ from Waelbroeck et al. (2002) subtracted from raw CB2 $\delta^{18}O_{speleothem}$ shown in black, to yield corrected CB2 $\delta^{18}O_{speleothem}$ shown in blue. Corrected $\delta^{18}O_{speleothem}$ was used in all figures, and all other speleothem records used for comparison were also corrected using values from Waelbroeck et al. (2002). Correcting our record for changes in mean seawater $\delta^{18}O$ does not affect the magnitude of change in millennial scale events. Furthermore, changes in $\delta^{18}O_{seawater}$ during the Holocene were minimal, reflected in the overlap of corrected (blue) and uncorrected (black) values.

Table S1. Uranium-thorium data for Stalagmite CB2

Depth (mm)	^{238}U (ng/g)	^{232}Th (ng/g)	$\delta^{234}U$ (‰)	$^{230}Th/^{238}U$ (activity)	$^{230}Th/^{232}Th$ (ppm atomic)	Uncorrected Age (yr)	Corrected Age (yr)	$\delta^{234}U_{init}$ (‰)
3	39 ± 1	339 ± 1	132 ± 2	0.1115 ± 0.0069	19.1 ± 1.2	11285 ± 735	5232 ± 3231	134 ± 2
14	38 ± 1	1438 ± 1	128 ± 3	0.0584 ± 0.0012	406.9 ± 10.2	5791 ± 123	5646 ± 143	130 ± 3
27	45 ± 1	2745 ± 1	129 ± 2	0.0611 ± 0.0019	271.9 ± 9.2	6061 ± 194	5833 ± 225	131 ± 2
45	52 ± 1	4552 ± 1	146 ± 2	0.1529 ± 0.0036	17.5 ± 0.4	15569 ± 394	6508 ± 4797	149 ± 3
85	63 ± 1	8563 ± 1	174 ± 2	0.1144 ± 0.0006	1648.9 ± 333	11153 ± 65	11085 ± 73	180 ± 2
90	59 ± 1	9059 ± 1	225 ± 3	0.1219 ± 0.0006	735.6 ± 7.5	11394 ± 66	11240 ± 102	232 ± 3
118	52 ± 1	11852 ± 1	191 ± 3	0.1255 ± 0.0006	290.9 ± 1.7	12107 ± 69	11694 ± 220	197 ± 3
149	51 ± 1	14951 ± 1	200 ± 2	0.1361 ± 0.0009	296.8 ± 2.8	13084 ± 95	12649 ± 240	207 ± 2
158	51 ± 1	15851 ± 1	213 ± 2	0.1401 ± 0.0008	874.6 ± 11.6	13337 ± 84	13186 ± 113	221 ± 2
179	50 ± 1	17950 ± 1	236 ± 2	0.1482 ± 0.0007	423.4 ± 2.9	13873 ± 74	13550 ± 179	245 ± 2
203	41 ± 1	20341 ± 1	240 ± 2	0.1529 ± 0.0007	1124.2 ± 16.9	14291 ± 74	14166 ± 97	250 ± 2
235	47 ± 1	23547 ± 1	230 ± 2	0.1623 ± 0.0008	730.4 ± 7.2	15364 ± 85	15159 ± 134	240 ± 2
314	22 ± 1	31422 ± 1	415 ± 2	0.2211 ± 0.0011	699.6 ± 113	18377 ± 103	18125 ± 164	437 ± 2
344	22 ± 1	34422 ± 1	447 ± 2	0.2649 ± 0.0017	154.2 ± 1	21822 ± 157	20480 ± 700	474 ± 2
376	30 ± 1	37630 ± 1	355 ± 3	0.2601 ± 0.0013	9453 ± 10.1	23026 ± 139	23004 ± 140	379 ± 3
382	22 ± 1	38222 ± 1	386 ± 2	0.2903 ± 0.0017	7273 ± 8.6	25349 ± 171	25316 ± 171	415 ± 2
413	18 ± 1	41318 ± 1	378 ± 6	0.3171 ± 0.0021	5283 ± 4.8	28172 ± 254	28123 ± 255	409 ± 7
447	27 ± 1	44727 ± 1	398 ± 2	0.3521 ± 0.002	157.6 ± 1	31196 ± 209	29397 ± 940	432 ± 2
483	39 ± 1	48339 ± 1	330 ± 2	0.3341 ± 0.0016	628 ± 3.9	31159 ± 179	30710 ± 289	360 ± 2
507	29 ± 1	50729 ± 1	313 ± 4	0.4449 ± 0.0065	53.4 ± 0.8	44324 ± 804	37024 ± 3907	347 ± 6
533	36 ± 1	53336 ± 1	318 ± 2	0.4241 ± 0.0026	92.6 ± 0.6	41638 ± 316	37700 ± 2052	354 ± 3
556	34 ± 1	55634 ± 1	313 ± 2	0.5263 ± 0.0074	39.5 ± 0.6	54594 ± 982	42748 ± 6432	353 ± 7
577	24 ± 1	57724 ± 1	298 ± 2	0.4789 ± 0.0034	81.6 ± 0.6	49237 ± 445	44103 ± 2696	337 ± 3
582	26 ± 1	58226 ± 1	297 ± 3	0.5013 ± 0.0039	60.1 ± 0.5	52183 ± 535	44815 ± 3897	337 ± 5
603	34 ± 1	60334 ± 1	270 ± 5	0.4674 ± 0.0044	1833 ± 1.7	49153 ± 628	48930 ± 638	310 ± 6
624	28 ± 1	62428 ± 1	263 ± 2	0.4901 ± 0.0029	79.4 ± 0.5	52541 ± 408	46976 ± 2917	300 ± 3
662	30 ± 1	66230 ± 1	254 ± 2	0.5019 ± 0.0031	87.6 ± 0.6	54672 ± 446	49482 ± 2725	292 ± 3
666	36 ± 1	66636 ± 1	238 ± 2	0.4819 ± 0.0025	129 ± 0.7	52824 ± 365	49416 ± 1787	274 ± 3
690	40 ± 1	69040 ± 1	286 ± 2	0.5011 ± 0.0019	316.1 ± 1.3	52750 ± 275	51378 ± 749	331 ± 2
709	49 ± 1	70949 ± 1	274 ± 2	0.5106 ± 0.0033	289.2 ± 1.9	54703 ± 465	53160 ± 912	318 ± 2
730	44 ± 1	73044 ± 1	240 ± 5	0.5071 ± 0.0037	388.5 ± 3	56248 ± 611	55075 ± 852	280 ± 6
754	34 ± 1	75434 ± 1	270 ± 1	0.6207 ± 0.0046	40.8 ± 0.3	71102 ± 729	57076 ± 7647	317 ± 7
774	31 ± 1	77431 ± 1	361 ± 3	0.6268 ± 0.0053	70.5 ± 0.6	65201 ± 761	57836 ± 3935	425 ± 6

All reported errors are 2σ. Errors for ^{238}U and ^{232}Th concentrations are estimated to be ±1% due to uncertainties in spike concentration; analytical uncertainties are smaller. Corrected ages are corrected for initial ^{230}Th assuming an initial $^{230}Th/^{232}Th$ of 10.5 ± 5.3 ppm. Decay constants for ^{230}Th and ^{234}U are from Cheng et al. (2013) decay constant for ^{238}U is 1.55125 x 10^{-10} yr^{-1} (Jaffey et al., 1971). All dates shown are in years before present (where present is 1950 CE).

Table S2. Radiocarbon data for a modern Cueva Bonita speleothem

Depth (mm)	Sample name	Fraction Modern	±	$D^{14}C$ (‰)	±	^{14}C age (BP)	±
4.5	Mexico_CB4_1	1.0456	0.0041	45.6	4.1	Modern	
5.75	Mexico_CB4_2	1.0733	0.0038	73.3	3.8	Modern	
6.5	Mexico_CB4_3	1.0887	0.0053	88.7	5.3	Modern	
7.5	Mexico_CB4_4	1.1447	0.0019	144.7	1.9	Modern	
8.5	Mexico_CB4_5	1.1060	0.0018	106.0	1.8	Modern	
9.5	Mexico_CB4_6	0.9040	0.0016	-96.0	1.6	810	15
10.5	Mexico_CB4_7	0.8843	0.0015	-115.7	1.5	990	15
11.5	Mexico_CB4_8	0.8901	0.0014	-109.9	1.4	935	15
12	Mexico_CB4_9	0.8906	0.0014	-109.4	1.4	930	15
12.5	Mexico_CB4_10	0.8771	0.0016	-122.9	1.6	1055	15

Radiocarbon results in the first 12.5 mm of speleothem sample CB4 demonstrating a rise in radiocarbon around 9.5 mm.

Table S3. Matching various initial Th to the bomb peak in a modern cave sample

$^{230}Th/^{232}Th$ Correction (ppm)	Depth (mm)	^{238}U (ng/g)	^{232}Th (ng/g)	$\delta^{234}U$ (‰)	$(^{230}Th/^{238}U)$ (Activity)	$^{230}Th/^{232}Th$ ppm atomic	Uncorrected Age (yr)	Corrected Age (yr before 2020)	$\delta^{234}U_{init}$ (‰)
4.4 ± 2.2	10	52 ± 1	165 ± 3	119 ± 2	0.0028 ± 0.0001	13.8 ± 0.53	269 ± 10	184 ± 44	119 ± 2
7.5 ± 3.8	10	52 ± 1	165 ± 3	119 ± 2	0.0028 ± 0.0001	13.8 ± 0.53	269 ± 10	123 ± 75	119 ± 2
10.5 ± 5.3	10	52 ± 1	165 ± 3	119 ± 2	0.0028 ± 0.0001	13.8 ± 0.53	269 ± 10	64 ± 104	119 ± 2
13.5 ± 7.6	10	52 ± 1	165 ± 3	119 ± 2	0.0028 ± 0.0001	13.8 ± 0.53	269 ± 10	6 ± 149	119 ± 2

Various initial Th values were tested at the same depth as the radiocarbon bomb peak and demonstrate an initial Th value of 10.5 is reasonable for Cueva Bonita.

Table S4: Precipitation from Alta Cima, Mexico and cave water $\delta^{18}O$ and δD data.

ID	Oxygen (VSMOW)	Hydrogen (VSMOW)	Date (m/d/yr)	Location	Type	Site
KW1	-1.31	2.74	5/14/18	Alta Cima	Precipitation	N/A
KW2	-2.29	-8.00	6/7/18	Alta Cima	Precipitation	N/A
KW3	-10.51	-72.17	6/21/18	Alta Cima	Precipitation	N/A
KW4	-14.03	-98.30	6/22/18	Alta Cima	Precipitation	N/A
KW5	-5.55	-33.62	6/24/18	Alta Cima	Precipitation	N/A
KW6	-10.28	-71.49	6/27/18	Alta Cima	Precipitation	N/A
KW7	-3.61	-16.12	6/28/18	Alta Cima	Precipitation	N/A
KW8	-1.25	-0.69	7/6/18	Alta Cima	Precipitation	N/A
KW9	-3.40	-12.97	7/7/18	Alta Cima	Precipitation	N/A
KW10	-4.99	-24.24	7/8/18	Alta Cima	Precipitation	N/A
KW11	-3.29	-10.32	8/5/18	Alta Cima	Precipitation	N/A
KW12	-3.97	-21.32	8/6/18	Alta Cima	Precipitation	N/A
KW13	-4.16	-25.34	8/12/18	Alta Cima	Precipitation	N/A
KW14	-0.85	6.72	8/13/18	Alta Cima	Precipitation	N/A
KW15	-1.46	-1.77	8/16/18	Alta Cima	Precipitation	N/A
KW16	-1.28	2.43	8/17/18	Alta Cima	Precipitation	N/A
KW17	-2.19	-3.02	8/25/18	Alta Cima	Precipitation	N/A
KW18	-2.74	-7.99	8/27/18	Alta Cima	Precipitation	N/A
KW19	-2.02	-1.01	8/28/18	Alta Cima	Precipitation	N/A
KW20	-2.64	-5.71	8/30/18	Alta Cima	Precipitation	N/A
KW21	-4.58	-22.23	9/7/18	Alta Cima	Precipitation	N/A
KW22	-4.48	-26.60	9/9/18	Alta Cima	Precipitation	N/A
KW23	-3.00	-9.69	9/10/18	Alta Cima	Precipitation	N/A
KW24	-4.33	-21.54	9/11/18	Alta Cima	Precipitation	N/A
KW25	-8.66	-53.86	9/13/18	Alta Cima	Precipitation	N/A
KW26	-6.31	-36.05	9/15/18	Alta Cima	Precipitation	N/A
KW27	-6.19	-35.53	9/16/18	Alta Cima	Precipitation	N/A
KW28	-2.13	-1.42	9/18/18	Alta Cima	Precipitation	N/A
KW29	-3.82	-15.74	9/21/18	Alta Cima	Precipitation	N/A
KW30	-3.91	-20.89	9/23/18	Alta Cima	Precipitation	N/A
KW31	-3.14	-9.97	9/27/18	Alta Cima	Precipitation	N/A
KW32	-3.17	-9.92	9/29/18	Alta Cima	Precipitation	N/A
KW33	-2.06	0.20	10/2/18	Alta Cima	Precipitation	N/A
KW34	-2.05	0.30	10/3/18	Alta Cima	Precipitation	N/A
KW35	-3.71	-12.92	10/7/18	Alta Cima	Precipitation	N/A
KW36	-3.56	-13.07	10/9/18	Alta Cima	Precipitation	N/A
KW37	-3.68	-12.96	10/11/18	Alta Cima	Precipitation	N/A
KW38	-3.70	-13.01	10/15/18	Alta Cima	Precipitation	N/A
KW39	-7.72	-48.65	10/16/18	Alta Cima	Precipitation	N/A
KW40	-7.57	-48.34	10/17/18	Alta Cima	Precipitation	N/A
KW41	-6.48	-37.64	10/26/18	Alta Cima	Precipitation	N/A
KW42	-4.64	-17.46	11/28/18	Alta Cima	Precipitation	N/A
KW43	-1.75	0.76	3/20/19	Alta Cima	Precipitation	N/A
KW44	-2.41	0.74	3/21/19	Alta Cima	Precipitation	N/A
KW45	-2.44	0.77	3/22/19	Alta Cima	Precipitation	N/A
KW46	-1.74	1.06	4/30/19	Alta Cima	Precipitation	N/A
KW47	0.22	-2.87	5/1/19	Alta Cima	Precipitation	N/A
KW48	-1.55	-2.54	5/3/19	Alta Cima	Precipitation	N/A
KW94	-2.85	-16.83	5/10/18	Cueva Bonita	Cave pool	D2-Pool
KW95	-4.10	-20.64	5/10/18	Cueva Bonita	Drip water	D1
KW96	-3.96	-20.36	5/10/18	Cueva Bonita	Drip water	D2
KW97	-3.89	-18.26	5/10/18	Cueva Bonita	Drip water	D3 - Multi
KW98	-3.05	-20.22	5/10/18	Cueva Bonita	Drip water	D4
KW99	-4.80	-23.27	5/10/18	Cueva Bonita	Drip water	D5
KW100	-5.50	-29.77	5/10/18	Cueva Bonita	Drip water	D6
KW101	-3.24	-15.36	5/10/18	Cueva Bonita	Drip water	Back room
KW118	-5.30	-27.77	5/4/19	Cueva Bonita	Drip water	D1
KW119	-3.51	-12.66	5/5/19	Cueva Bonita	Drip water	D2
KW120	-4.27	-19.30	5/6/19	Cueva Bonita	Cave pool	D2-Pool
KW121	-5.11	-26.52	5/7/19	Cueva Bonita	Drip water	D4
KW122	-3.44	-12.64	5/8/19	Cueva Bonita	Drip water	D5
KW123	-4.18	-18.53	5/9/19	Cueva Bonita	Drip water	D6
KW124	-5.47	-29.54	5/10/19	Cueva Bonita	Drip water	D7
