# Supplementary material for: Dynamic and thermodynamic influences on precipitation in Northeast Mexico on orbital to millennial timescales
Source: Nat Commun. 2023 Apr 20;14:2279. doi: 10.1038/s41467-023-37700-9 (PMC10119167; doi:10.1038/s41467-023-37700-9)
Supplement: Supplementary file 3 — Description of Additional Supplementary Files [file 41467_2023_37700_MOESM3_ESM.pdf]

File name: Supplementary Data 1

Description: Stable isotope and trace element data.
